# The rise of proteome‐wide biophysics

**DOI:** 10.15252/msb.202110442

**Published:** 2021-07-22

**Authors:** Andre Mateus, Mikhail M Savitski, Ilaria Piazza

**Affiliations:** ^1^ Genome Biology Unit European Molecular Biology Laboratory Heidelberg Germany; ^2^ Max Delbrück Center for Molecular Medicine in the Helmholtz Association (MDC Berlin) Berlin Germany

## Abstract

While informative, protein amounts and physical protein associations do not provide a full picture of protein function. This Commentary highlights the potential of structural and stability proteomic technologies to derive new insights in biology and medicine.

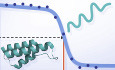

The ultimate goal of proteomics is to provide a holistic view of the biological processes that explain phenotypes. To this end, proteomics approaches have been frequently used to map protein expression in multiple experimental conditions and to identify protein–protein interactions. While informative to a certain extent, protein amounts and physical protein associations do not provide a full picture of protein function. Here, we highlight the novel insights that are made possible in biology and medicine by structural and stability proteomic technologies.

## From protein expression profiles to protein biophysical profiles

Understanding the relationship between genotypes and phenotypes is one of the open challenges in life sciences. Since proteins orchestrate all cellular processes, their systematic study in the cellular context is a powerful strategy for dissecting cellular phenotypes. Many research groups have studied the fundamentals of protein cellular networks by cataloguing proteins differentially expressed in tissues, cells and organelles. Mass spectrometry (MS)‐based methods can detect close to 90% of annotated protein‐coding genes, achieving almost complete coverage of the human cell proteome. This comprehensive protein identification combined with precise quantification of protein abundance makes it possible to map which genetic functional units are required in different cellular contexts. While the expression proteomics strategy has been successful in capturing a subset of functional changes in biological systems, a large part of biological regulation does not manifest itself in changes in protein level.

More than a decade ago, the term “*functional proteomics*” was introduced to describe the early attempts to characterize the complexity of protein‐related molecular events and their functional consequences. Until recently, this concerted effort has mostly focused on the identification of protein–protein interactions, as physical associations among proteins suggest their involvement in a common biological function (*guilt by association*). However, protein activity is not only affected by protein–protein interactions, but it is also dependent on interactions with small molecules, cofactors, metals, and nucleic acids, as well as on the folding state (e.g. aggregation, stability or misfolding). All these factors combined with the local cellular environment (e.g. pH, osmotic pressure, temperature) result in alterations of the *biophysical properties* of proteins that ultimately influence biological processes.

The fundamental principle that protein structure defines the function of proteins derives from single‐protein studies in diluted solutions. *In vitro* assays provide clear mechanistic insights about structure–activity associations but they are limited in throughput and do not necessarily represent the behaviour of proteins in cells. Recently developed proteomic‐based methods allow the global characterization of protein structure and interaction states under near‐physiological conditions, unifying reductionist and systematic biophysical approaches. These include **t**hermal **p**roteome **p**rofiling (TPP) (Savitski *et al*, 2014) and **li**mited **p**roteolysis coupled with **m**ass **s**pectrometry (LiP‐MS; Feng *et al*, 2014), which allow global structural readouts that can be directly linked to protein function *in situ,* thus showing a crucial increase of the scope of classical proteomics technologies.

## Expanding the functional proteomics landscape with biophysics proteomics

**Figure 1:**
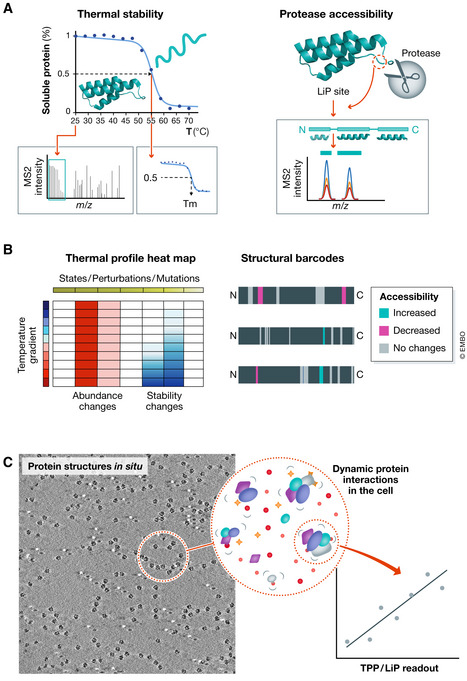
Biophysics proteomics: principles and potential applications of thermal proteome profiling (TPP) and limited proteolysis coupled with mass spectrometry (LiP‐MS) (A) In TPP, cells or lysates are heated in a range of different temperatures obtaining thermal stability curves from the fraction of soluble proteins present in the mixture. The denaturation temperature is an intrinsic property of each protein and it is described by the melting temperature (*Tm*), which is the temperature when half of the protein is denatured and appears as an inflection point in the melting curve. Soluble proteomes are quantified using multiplexed quantitative proteomics. In LiP‐MS, sterically accessible sites of protein structures (*LiP‐sites*) are cleaved by proteases, the resulting peptide patterns are measured by bottom‐up proteomics obtaining a LiP fingerprint of protein structural features. (B) Graphical representations of protein stability (*thermal profile heat map*) and protein structural features (*structural barcodes*) for each protein measured in TPP and LiP‐MS experiments. (C) Integration of *in situ* structural biology methods (cryo‐ET) that visualize protein molecular machines in the cellular context with biophysics proteomics technologies: biophysics proteomics gives additional information about protein interactions and functional states to high‐resolution structural models of the cell.

The principles of TPP and LiP‐MS are similar: they both use readouts of protein structures measurable for thousands of proteins at the same time with mass spectrometry. The technologies are complementary with LiP‐MS providing hints on where a structural alteration takes place along the protein sequence and TPP enabling the readout of protein thermal stability from intact cells and tissues. Moreover, since LiP‐MS and TPP employ different biophysical concepts, their combined use can significantly improve the structural characterization of proteomes. LiP‐MS provides a snapshot of all proteins that undergo structural changes using controlled proteolytic digestions. The resulting peptide patterns are a reflection of the protein shape and differential accessibility to the protease. In TPP the soluble protein fraction is measured over a temperature gradient as proxy of the stably folded proteins at a given temperature (Fig 1A and B). Variations of the proteolytic fingerprint in LiP or a change in the temperature at which the denaturation occurs (i.e. its thermal stability) in TPP may be due to protein interactions with other molecules or post‐translational modifications in the cellular *milieu*. Systematic analyses in large‐scale datasets have found evidence of multiple associations between protein structural and stability readouts and expected molecular interaction events occurring *in vivo*.

A recent TPP study explored the relationship between genetic perturbations (121 deletions in *E*. *coli*) and protein thermal stability parameters *in vivo*. In analogy with the principle that functionally related genes are co‐expressed, proteins with coordinated changes of thermal stability are involved in the same biological processes (Mateus *et al*, 2020). For instance, enzymes in a metabolic network, the subunits of protein complexes and components of operons define clusters of proteins with similarly altered levels and thermal stability in different mutant strains. As a result, identifying co‐melting partners of orphan genes can reveal their unknown function in a *guilt by association* manner.

With LiP‐MS, these association analyses are more difficult to contextualize, since peptides mapping on the same protein provide independent structural information. However, the structural resolution of LiP‐MS is higher than TPP and focused at the protein domain level. LiP signals that are localized in active sites of metabolic enzymes or protein kinases are associated with metabolite binding events with regulatory function. Structural changes measured with LiP peptides correlate with known variations of activity of signalling pathways, phosphorylation events or metabolic fluxes and are therefore a proxy of functionally relevant events (Cappelletti *et al*, 2021). As more LiP‐MS and TPP datasets profiling different processes and diseases become available, we envision the use of machine‐learning approaches to identify which structural features of proteins have regulatory relevance and, in the long term, to prioritize functional sites in a context‐specific manner. Connecting proteomics data with functional information using machine learning is feasible, as recently demonstrated for the prediction of functional phosphorylation events in proteins, with functional sites more commonly leading to changes in protein thermal stability.

Importantly, the information provided by the variation of protein abundance and protein stability is largely orthogonal. Housekeeping genes, whose expression is necessary for survival, are in general more frequently involved in physical interactions between proteins or modulated by small molecules than genes whose expression levels are heavily controlled at the level of synthesis or degradation (Piazza *et al*, 2018; Mateus *et al*, 2020). The expression of conditional genes allows the cell to perform new functions, while structural changes of proteomes adjust cellular processes through allostery. Therefore, TPP and LiP‐MS are useful tools to characterize those genes that do not change their mRNA or protein levels and as a result are inaccessible with standard proteomics and transcriptomics assays. Similarly, detecting correlative relationships between global protein structural parameters and gene ontologies can inform about the function of essential genes that are difficult to study with reverse genetics screens.

## Dynamic protein interactions in the cellular *milieu*

Analyses based on correlations are powerful, because they can detect various types of protein molecular interactions simultaneously. However, sometimes the focus of an investigation may be a specific mechanism causing structural alterations. In those cases, it is possible to design specific LiP‐MS and TPP assays to discriminate whether protein complex formation, post‐translational modifications or enzymatic reactions are involved.

For instance, proteome‐wide structural changes can identify protein–small molecule interaction events when cells are exposed to a single compound in a controlled experimental set‐up. With this assay design, the candidate protein targets of the small molecule change their melting temperature (*Tm*) or alter their protease sensitivity (*LiP fingerprint*). Thermal stability workflows have been developed for target identification of different drug classes, including antibiotics, and can be applied to intact eukaryotic and prokaryotic cells, mammalian tissues and can map transmembrane targets. Notably, TPP experiments performed on whole cells determine which protein targets are engaged *in situ*, while experiments performed in lysates can help deconvolute direct targets from downstream effects. In addition, using natural compounds instead of drugs, it is possible to discover new metabolite binding proteins. With LiP‐MS, the position of proteolytic cleavages provides hints for identifying small molecule binding sites, bringing the resolution of structural proteomics methods to single compound binding sites (Piazza *et al*, 2018, 2020). Refined strategies for TPP and LiP‐MS data analysis are now employing functional statistics and machine learning to reduce false‐positive rates, making the systematic mapping of protein–ligand interactions more robust.

Biophysical proteomics methods are also suitable to study protein–protein interactions based on the simple rationale that protein‐forming complexes have often similar thermal stabilities, thus co‐aggregate or are in general more protected from proteolysis. Searching for clusters of co‐melting proteins has a unique advantage over other protein–protein interaction analysis methods, as this strategy does not require crosslinking labelling, co‐elution or affinity purification. In analogy to the applications for protein–small molecule interactions, biophysical proteomics methods are performed in native conditions that maintain protein structures and avoid introducing confounding effects (Tan *et al*, 2018). The dynamic assembly of protein complexes can also be monitored by finding proteins that undergo structural changes when cellular lysates are exposed to different perturbations.

## The big picture of protein folding

The functional proteome of an organism defines the ensemble of chemical reactions and interactions of the cell that occur when proteins are properly folded. The environmental conditions when protein folding can be successfully completed are therefore as important as the linear protein sequence in defining protein functions. Recently, applications measuring the fraction of soluble proteins or LiP fingerprint readouts have addressed the problem of how proteins unfold upon temperature (Leuenberger *et al*, 2017; Sridharan *et al*, 2019). TPP meltomes of archaea, bacteria and human species have provided evidence that the melting temperature of a proteome is shifted towards the range of optimal growing temperature of an organism and explain why loss of proteome function can be a main cause of cell death after severe heat stress. Global meltome analyses further revealed that evolutionary conservation of protein complexes is reflected by similar thermal stability of their subunits (Jarzab *et al*, 2020). Peptide melting curves derived from LiP data from distinct protein domains have distinct melting temperatures and define measurable independently folded units within the cell (Leuenberger *et al*, 2017). In the future, these types of data will further help in finding the essential factors controlling protein folding efficiency, such as the contribution of protein chaperones and environmental conditions.

## Global analysis of protein aggregates and the concept of structural biomarkers

TPP and LiP‐MS are also useful to study protein aggregation‐like events, providing a proteome‐wide perspective to the analysis of protein solubility transition or of phase separated organelles upon stress and cell states. For instance, some human proteins decrease in solubility and some bacterial proteins form oligomers upon ATP binding, suggesting a role for ATP in driving protein condensation transitions (Piazza *et al*, 2018; Sridharan *et al*, 2019). With LiP‐MS, it is also possible to detect soluble‐to‐aggregate structural changes of the alpha‐synuclein protein, potentially enabling a new understanding of protein aggregation disorders (Feng *et al*, 2014). This work illustrates how scientists could use new protein structure centred ‐*omics* approaches to gain new insights into neurodegenerative disorders, where the pathogenesis of the disease is caused by protein mislocalization or fibrillation and not by protein expression changes. Increasing the throughput of protein biophysical methods and their applicability to biological fluids such as plasma could pave the way for defining structural biomarkers for the early diagnosis of pathogenic proteinopathies.

## *In* *situ* structural biology… with *‐omics*

Genome‐wide protein biophysics explores a new dimension of the proteome that makes structural biology operational on the *‐omics* level. Although biophysical proteomics provides systematic information about dynamic structural changes directly linkable to protein functions, it lacks the genuine atomic resolution of classical *in vitro* structural studies. How can we then zoom into the fine molecular details of protein structures while keeping a system‐wide prospective?

The direct visualization of protein molecular machines with sub‐nm resolution in the context of cellular architectures, organelles or condensates is now possible with the latest advances in cryo‐electron tomography (cryo‐ET) or super‐resolution microscopy. The present challenge is thus to come up with ways to monitor the dynamics of native protein structures at high resolution over time, including protein structural changes occurring due to interactions with small molecules, nucleic acids and the formation of condensates. We can envision a new form of protein structural analysis that integrates static images obtained from electron tomograms with biophysical parameters of proteins measured on a cell‐wide scale with mass spectrometry (Fig 1C). For instance, the dissolution of protein complexes or phase separated compartments that would manifest with a change of protein stability would also be captured in sequential snapshot images acquired with microscopy *in situ*.

Stability proteomics and cryo‐ET are complementary. They can elegantly link endogenous interactions to molecular machines by visualizing variations of localization and structure of protein assemblies upon changes of the cellular *milieu*. Likewise, *in situ* structural models from cryo‐ET provide a scaffold for annotating different kinds of interactions from native structures. We discussed the application of LiP‐MS and TPP technologies for the unbiased capturing of protein state changes (caused by post‐translational modifications, cofactors, ligand binding and interaction with other proteins). However, in‐cell crosslinking mass spectrometry can also add valuable modelling information relating covalently linked sites to their proximity in protein complexes. Even more excitingly, these new integrative microscopy‐proteomics workflows could become a routine way of including high‐resolution protein 3D structures into network models of metabolism, gene expression and proteostasis.

## Conflict of interest

The authors declare that they have no conflict of interest.

## Further reading

Functional protein phosphorylation

Huang
JX

*et al* (2019) High throughput discovery of functional protein modifications by Hotspot Thermal Profiling. Nat Methods
16: 894–901
3138404310.1038/s41592-019-0499-3PMC7238970

Ochoa
D
, 
Jarnuczak
AF
, 
Viéitez
C
, 
Gehre
M
, 
Soucheray
M
, 
Mateus
A
, 
Kleefeldt
AA
, 
Hill
A
, 
Garcia‐Alonso
L
, 
Stein
F

*et al* (2019) The functional landscape of the human phosphoproteome. Nat Biotechnol
38: 365–373
3181926010.1038/s41587-019-0344-3PMC7100915

Potel
CM
, 
Kurzawa
N
, 
Becher
I
, 
Typas
A
, 
Mateus
A
, 
Savitski
MM
 (2021) Impact of phosphorylation on thermal stability of proteins. Nat Methods
18: 1–3
3414070010.1038/s41592-021-01177-5

Smith
IR
, 
Hess
KN
, 
Bakhtina
AA
, 
Valente
AS
, 
Rodríguez‐Mias
RA
, 
Villén
J
 (2021) Identification of phosphosites that alter protein thermal stability. Nat Methods
18: 760–762
3414069910.1038/s41592-021-01178-4PMC8783534Data analysis

Childs
D
, 
Bach
K
, 
Franken
H
, 
Anders
S
, 
Kurzawa
N
, 
Bantscheff
M
, 
Savitski
MM
, 
Huber
W
 (2019) Nonparametric analysis of thermal proteome profiles reveals novel drug‐binding proteins. Mol Cell Proteomics
18: 2506–2515
3158255810.1074/mcp.TIR119.001481PMC6885700

Kurzawa
N
, 
Becher
I
, 
Sridharan
S
, 
Franken
H
, 
Mateus
A
, 
Anders
S
, 
Bantscheff
M
, 
Huber
W
, 
Savitski
MM
 (2020) A computational method for detection of ligand‐binding proteins from dose range thermal proteome profiles. Nat Commun
11: 5783
3318819710.1038/s41467-020-19529-8PMC7666118

Kurzawa
N
, 
Mateus
A
, 
Savitski
MM
 (2020b) Rtpca: an R package for differential thermal proximity coaggregation analysis. Bioinformatics
37: 431–433
10.1093/bioinformatics/btaa682PMC805877632717044

Piazza
I
, 
Beaton
N
, 
Bruderer
R
, 
Knobloch
T
, 
Barbisan
C
, 
Chandat
L
, 
Sudau
A
, 
Siepe
I
, 
Rinner
O
, 
de Souza
N

*et al* (2020) A machine learning‐based chemoproteomic approach to identify drug targets and binding sites in complex proteomes. Nat Commun
11: 4200–4213
3282691010.1038/s41467-020-18071-xPMC7442650

Schopper
S
, 
Kahraman
A
, 
Leuenberger
P
, 
Feng
Y
, 
Piazza
I
, 
Müller
O
, 
Boersema
PJ
, 
Picotti
P
 (2017) Measuring protein structural changes on a proteome‐wide scale using limited proteolysis‐coupled mass spectrometry. Nat Protoc
12: 2391–2410
2907270610.1038/nprot.2017.100Applications in bacteria, cells and tissues

Becher
I
, 
Andrés‐Pons
A
, 
Romanov
N
, 
Stein
F
, 
Schramm
M
, 
Baudin
F
, 
Helm
D
, 
Kurzawa
N
, 
Mateus
A
, 
Mackmull
M‐T

*et al* (2018) Pervasive protein thermal stability variation during the cell cycle. Cell
173: 1495–1507
2970654610.1016/j.cell.2018.03.053PMC5998384

Dai
L
, 
Zhao
T
, 
Bisteau
X
, 
Sun
W
, 
Prabhu
N
, 
Lim
YT
, 
Sobota
RM
, 
Kaldis
P
, 
Nordlund
P
 (2018) Modulation of protein‐interaction states through the cell cycle. Cell
173: 1481–1494
2970654310.1016/j.cell.2018.03.065

Kalxdorf
M
, 
Günthner
I
, 
Becher
I
, 
Kurzawa
N
, 
Knecht
S
, 
Savitski
MM
, 
Eberl
HC
, 
Bantscheff
M
 (2021) Cell surface thermal proteome profiling tracks perturbations and drug targets on the plasma membrane. Nat Methods
18: 84–91
3339819010.1038/s41592-020-01022-1

Mateus
A
, 
Bobonis
J
, 
Kurzawa
N
, 
Stein
F
, 
Helm
D
, 
Hevler
J
, 
Typas
A
, 
Savitski
MM
 (2018) Thermal proteome profiling in bacteria: probing protein state in vivo. Mol Syst Biol
14: e8242–15
2998061410.15252/msb.20188242PMC6056769

Perrin
J
, 
Werner
T
, 
Kurzawa
N
, 
Rutkowska
A
, 
Childs
DD
, 
Kalxdorf
M
, 
Poeckel
D
, 
Stonehouse
E
, 
Strohmer
K
, 
Heller
B

*et al* (2020) Identifying drug targets in tissues and whole blood with thermal‐shift profiling. Nat Biotechnol
38: 303–308.3195995410.1038/s41587-019-0388-4
